# Microbial synthesis of bimetallic Pd–Rh and Pd–Pt nanoparticle catalysts

**DOI:** 10.1039/d5na00861a

**Published:** 2025-12-16

**Authors:** Jinxin Xie, Christopher Egan-Morriss, Victoria S. Coker, Sam Sullivan-Allsop, Rongsheng Cai, Sarah J. Haigh, Jonathan R. Lloyd

**Affiliations:** a Department of Earth and Environmental Sciences, The University of Manchester Manchester UK jon.lloyd@manchester.ac.uk jinxin.xie@manchester.ac.uk christopher.eganmorriss@manchester.ac.uk; b Department of Materials, University of Manchester Oxford Road Manchester M13 9PL UK

## Abstract

In this study the metal-reducing bacterium, *Geobacter sulfurreducens*, was used to efficiently recover palladium (Pd), platinum (Pt), and rhodium (Rh) from solution *via* enzymatic bioreduction to form monometallic or bimetallic bio-PGM nanoparticles. Herein, we report the novel biosynthesis of bimetallic PdRh alloy nanoparticles (bio-PdRh), along with bimetallic PdPt nanoparticles (bio-PdPt). In monometallic solutions, *G. sulfurreducens* biosynthesised Pd(0), Pt(0), and Rh(0) nanoparticles supported at the cell surface, consistent with bioreduction by outer membrane c-type cytochromes. However, in bimetallic solutions, the cells preferentially bioreduced Pt(iv) over Pd(ii), resulting in Pt-rich bio-PdPt nanoparticles and highly dispersed Pd(ii) cell-surface clusters. In contrast, co-bioreduction of Pd(ii) and Rh(iii) led to the formation of PdRh alloy nanoparticles. We hypothesise that differences in the reduction potentials of the metal complexes were key to forming these different nanostructures. The reduction of 4-nitrophenol was used to assess bionanoparticle catalytic activity. Monometallic bio-Pt and bio-Rh displayed low activity for this reaction, whereas bio-Pd nanoparticles were highly active and gave the fastest initial reaction rate. Bimetallic bio-PdPt and bio-PdRh catalysts performed comparably to bio-Pd, using half the Pd content. This work highlights the ability of metal-reducing bacteria to synthesise functional nanocatalysts while recovering precious metals from mixed metal-containing wastewaters.

## Abstract 2

Microbial recovery of Pd, Pt, and Rh enables the green biosynthesis of bimetallic PdPt and PdRh nanoparticles, including novel PdRh alloys, which show high catalytic activity for 4-nitrophenol reduction.

## Graphical abstract

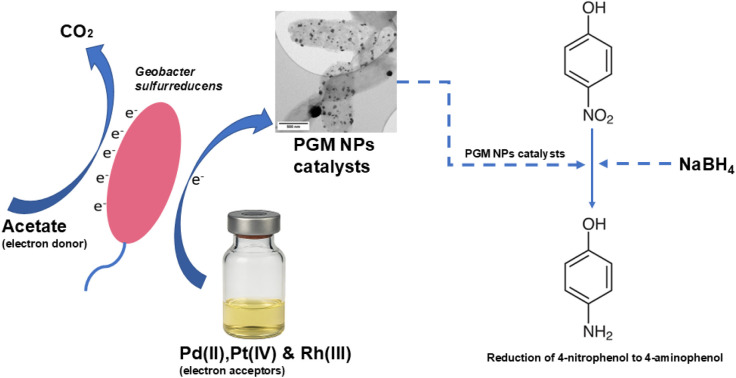

## 1. Introduction

The six platinum group metals (PGM) are platinum (Pt), palladium (Pd), rhodium (Rh), ruthenium (Ru), iridium (Ir) and osmium (Os). Of these, Pt, Pd and Rh are most significant in terms of global supply and demand due to their extensive use as automotive catalysts, as well as in chemical syntheses, fuel cells, electronic components, jewellery, medicine and dentistry, and as investment commodities.^1^ PGMs are among the least abundant elements in the Earth's crust and economic ore deposits,^2^ and therefore production occurs in just a few countries; primarily in the Bushveld Igneous Complex, South Africa, with deposits also present in Russia, Canada, Zimbabwe and the United States.^3^ The PGM supply chain is thus highly vulnerable and this contributes towards volatile prices and the status of PGMs as critical raw materials.^4^ The efficient recovery and recycling of PGMs from secondary sources is therefore essential to create stable and secure PGM availability to supply decarbonising technologies for the green transition away from fossil fuels, as well as preventing environmental PGM contamination.

The biorecovery of critical and scarce metals by microorganisms has been explored as a sustainable, economical and scalable alternative to conventional chemical recycling techniques.^5^ The microbial bioreduction of high oxidation state PGMs from aqueous solution is a promising biorecovery strategy, which can facilitate precious metal recovery while generating value-added nanoparticle catalysts.^6^ During the bioreduction process, precious metal ions are bound by microbial cell surface functional groups and electrons are enzymatically transferred to the metal, resulting in the immobilisation of cell-supported metal nanoparticles. Studies have investigated the bioreduction of Pd(ii) from single metal synthetic solutions, demonstrating the importance of biosynthesis parameters such as microbial species, metal loading, electron donor, and solution chemistry on bionanoparticle catalytic properties such as hydrogenation and C–C coupling reactions.^7–9^ The bioreduction of Pt(iv) and Pt(ii) to bio-Pt(0) nanoparticles has also been studied widely in bacteria, with bio-Pt proving to be catalytically active for selective hydrogenation.^10^ The bioreduction of Rh(iii) to bio-Rh(0) was demonstrated using *Shewanella algae*, and a sulfate-reducing bacterial consortium, however no catalytic performance tests were performed on bio-Rh.^11^

Bimetallic nanoparticles can exhibit both the combined properties of the two monometallic components, as well as new properties due to synergistic effects, resulting in enhanced catalytic performance compared to equivalent single metal catalysts.^12^ The structure of bimetallic nanoparticles is key to determining their optical, catalytic, and photocatalytic properties; structures can range from separate particles to mixed or random alloys, core–shell structures, or particles made up of segregated subclusters that share a mixed interface.^13^ Precise synthesis of bimetallic nanoparticles can control the size, shape, composition and morphology, which is highly desirable to fine-tune catalytic properties.^14^ The standard reduction potential (*E*^0^, V *vs.* the standard hydrogen electrode (SHE)) of a metal ion provides a reference for how easily it is reduced, and in general similar reduction potentials can yield alloy-type structures whereas widely differing reduction potentials can produce heterogeneous structures.^15^ The PGMs typically follow the order of reduction Pt > Pd > Rh, and are known to undergo galvanic replacement reactions, where one metal transfers electrons to a second metal with higher reduction potential.^16^ Pd-based bimetallic nanoparticles have received considerable attention in catalysis for petrochemical industrial processes, fine chemical synthesis, environment protection, and renewable energy conversion, as their specific activity and selectivity are usually higher than that of monometallic Pd.^17^ Combinations of bimetallic Pd, Pt and Rh make up three-way automotive catalysts,^18^ and bimetallic PdRh and PdPt catalysts have been investigated for transfer and selective hydrogenations,^19,20^ and for electrocatalysis such as the hydrogen evolution reaction^21^ and formate oxidation reaction.^22^

Recently, Pd-based bimetallic nanoparticles such as PdAu, PdAg, PdPt, and PdRu were microbially synthesised using the Gram-negative bacteria *Escherichia coli*, *Desulfovibrio desulfuricans*, and *Shewanella oneidensis*, and the Gram-positive bacterium *Bacillus benzeovorans*, which all displayed enhanced catalytic performance over their monometallic counterparts.^23,24^ Previous work has also found that co-bioreduction of Pd(ii) and Au(iii), *i.e.* microbial cells challenged with both metals at the same time, produced nanoparticles with enhanced catalytic performance compared to PdAu particles produced from sequential bioreduction.^25^ In this study, the metal-reducing bacterium *Geobacter sulfurreducens* was utilised to produce bimetallic PdPt and PdRh nanoparticles *via* the co-bioreduction of Pd(ii) and Pt(iv) or Rh(iii). The nanoparticles generated were analysed by a suite of techniques including X-ray Diffraction (XRD), Transmission Electron Microscopy (TEM) and X-ray Photoelectron Spectroscopy (XPS). PGM nanoparticles were tested for catalytic activity using the model 4-nitrophenol reduction reaction. Enhancing the understanding of bimetallic PGM nanoparticle formation is highly relevant to the development of novel bio-based processes for PGM biorecovery and tailored bio-metallic nanocatalyst formation from real-world mixed metal liquid effluents, with the potential to improve resource supply while reducing associated environmental waste.

## 2. Materials and methods

### 2.1 Cultivation of *Geobacter sulfurreducens*

An anaerobic bacterial growth medium containing sodium acetate (20 mM) as the electron donor and sodium fumarate (40 mM) as the electron acceptor was prepared.^26^*G. sulfurreducens* was incubated at 30 °C in the growth medium until late log phase growth prior to being harvested. All manipulations were performed under an 80 : 20 mix of N_2_ : CO_2_ gas to maintain anoxic conditions. The culture was harvested through centrifugation at 4920*g* and 4 °C for 20 minutes and washed with 30 mM sodium bicarbonate (NaHCO_3_; pH7) twice. Harvested cells were then resuspended in NaHCO_3_ solution and flushed with an 80 : 20 mix of N_2_ : CO_2_ before use in the bioreduction experiments.

### 2.2 Bioreduction of PGMs

Stock solutions (50 mM) of sodium tetrachloropalladate(ii) (Na_2_PdCl_4_), sodium hexachloroplatinate(iv) (NaPtCl_6_), and rhodium(iii) chloride hydrate (RhCl_3_·*x*H_2_O) were prepared in deionised water (DIW) and stored at 4 °C. An aliquot of PGM stock solution (final concentration 200 µM), sodium acetate (10 mM) and sodium bicarbonate (30 mM) were added to serum bottles to a final volume of 10 ml. An 80 : 20 mix of N_2_ : CO_2_ was used to flush each bottle for at least 0.5 hours, maintaining the pH at 6.8–7.0. Resting cells of *G. sulfurreducens* were added to each bottle to achieve a final OD_600_ of 0.4. Controls either without cells or without an electron donor were also set up. All bottles were incubated at 30 °C and sampled at 1 hour, 2 hours, 4 hours, and 24 hours. For mixed PGM bioreduction experiments, 100 µM of either Pt, or Rh was added to bottles containing 100 µM Pd. The above biological reduction experiment was repeated for the mixed PGM experiments.

Upon reaction completion the bionanoparticles were washed several times with DIW and resuspended in 2 ml DIW as a concentrated slurry. 100 µL aliquots of each bio-PGM sample were taken and digested in aqua regia for 6 hours at 60 °C, these samples were then used to quantify the PGM concentrations of bio-PGM suspensions using ICP-MS analysis.

### 2.3 Chemical and solid-phase analyses

PGM concentrations in solution were quantified using an Agilent 8800 Triple Quadrupole Inductively coupled plasma mass spectrometer (ICP-MS). At each monitoring point, 0.3 ml of solution was sampled and centrifuged at 14800*g* for 20 minutes. After centrifuging, 0.1 ml of supernatant was acidified with 9.9 ml 2% nitric acid (HNO_3_) for ICP-MS analysis. The prepared samples were stored in a cold room before analysis. The pH of the samples was also measured.

XRD was performed on a Bruker D2 Phaser diffractometer operated at 30 kV and 30 mA with a Cu X-ray source (*λ* = 1.5406 Å). The scan range was 5–70° 2*θ* with a step size of 0.04° and 0.4 s per step. EVA version 5 with the ICDD (International Centre for Diffraction Data) Database was used to process the XRD results. Approximately 3 ml of each PGM sample was taken and centrifuged at 14800*g* for 20 minutes to collect the precipitate, which was washed in DIW twice to remove salts. The washed precipitates were mixed with 1 ml amyl acetate and transferred to a low background Si wafer and air dried before XRD scanning.

XPS was performed using an Axis Ultra Hybrid spectrometer (Kratos Analytical, Manchester, United Kingdom) using monochromated Al Kα radiation (1486.6 eV, 10 mA emission at 150 W, spot size 300 × 700 µm) with a base vacuum pressure of ∼5 × 10^(−9)^ mbar. Charge neutralisation was achieved using a filament. Binding energy scale calibration was performed using C–C in the C 1s photoelectron peak at 284.8 eV. High resolution scans were performed on the Pd 3d, Pt 4f, and Rh 3d regions. Analysis and curve fitting was performed using Voigt-approximation peaks, fitted with a Shirley background using CasaXPS. Around 10 ml of the PGM solution was centrifuged at 14800*g* for 20 minutes and washed twice with DIW, then dried onto a silicon wafer.

TEM images were collected on a Tecnai T20 with 200 kV accelerating voltage. Scanning Transmission Electron Microscopy (STEM) was performed on a FEI Titan G2 80–200 S/TEM “ChemiSTEM™” instrument operated at 200 kV, equipped with a 0.7 mrad solid angle Energy Dispersive X-ray Spectroscopy (EDS) detector and a high angle annular dark field (HAADF) detector operated with a STEM convergence semi-angle of 21 mrad and a HAADF inner semi-angle of 55 mrad. Image and EDS spectral analysis was performed using custom Python scripts developed in-house, employing several publicly available packages, including hyperspy,^27^ skimage,^28^ and particlespy.^29^ The image preprocessing encompassed the application of a rolling ball-type background subtraction from the skimage Python package. Following this, segmentation and watershed thresholding were performed on the background-subtracted HAADF images, extracting particle locations and sizes. The resulting watershed thresholded image served as a mask for the corresponding EDS spectral image, enabling us to determine quantitative PGMs compositions using the standardless Cliff-Lorimer factor approach.

PGM samples were collected and centrifuged at 14 800 rpm for 30 minutes, washed three times with DIW, diluted and resuspended in DIW before drop-casting onto a copper grid coated with an amorphous carbon film. Size analysis was performed from TEM or HAADF-STEM images using ImageJ software, measuring two perpendicular diameter measurements each for roughly 60 particles per sample.

### 2.4 Catalytic experiments

Typically, 0.03 ml of 10 mM 4-nitrophenol solution and 1 ml of 60 mM NaBH_4_ were added into a quartz cuvette containing 1.97 ml of DIW. To initiate the reaction, the required volumes of bio-PGM, or cell only, suspensions were pipetted into the above solution to give a 4 mol% loading of metal catalyst, defined as total PGM moles (Pd + Pt/Rh) relative to the initial 4-NP moles. The cuvette holder was placed onto an orbital shaker at 100 rpm. The reaction was performed at room temperature and was recorded using a UV-vis spectrophotometer (Bruker), with spectra collected in the range 200–600 nm at 0, 0.75, 1.5, 3, 5, 10, 20, 40, and 60 minutes. The concentration of 4-nitrophenol was determined from the absorbance peak at 400 nm, for which a standard calibration curve was determined from the 4-nitrophenol stock solution. Under excess NaBH_4_, the reduction of 4-nitrophenol was treated as pseudo first order in 4-NP. The apparent rate constant, *k* (min^−1^), was obtained by linear regression of ln(*A*_t_/*A*_0_) at 400 nm over the initial linear region.

## 3. Results and discussion

### 3.1 Biorecovery of PGMs from solution

#### 3.1.1 Biorecovery from monometallic PGM solutions

**Fig. 1:**
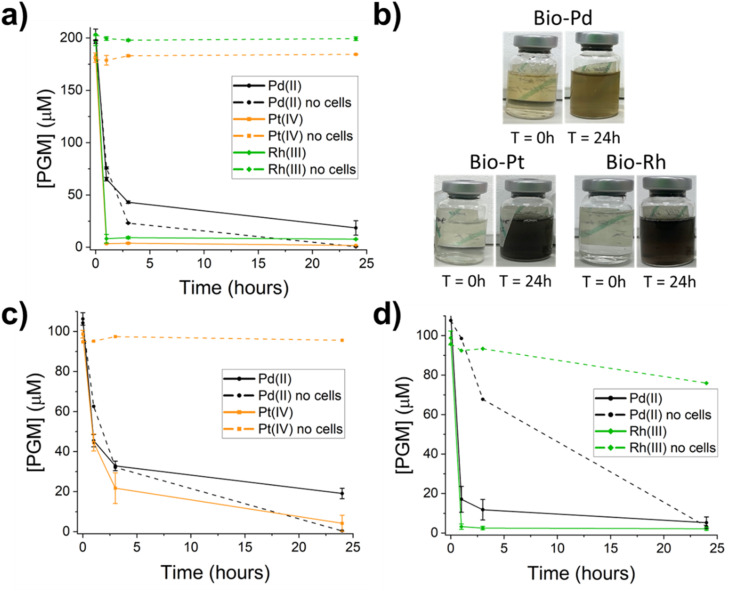
Changes in aqueous concentration of PGMs during bioreduction experiments with *G. sulfurreducens*. (a) The removal of PGMs in the presence and absence of cells from monometallic solutions of Pd, Pt, and Rh. (b) Images showing the change in colour of single metal solutions between the start (0 hours) and end (24 hours) of reactions. (c and d) Changes in PGM concentrations in bimetallic solutions in the presence and absence of cells for (c) PdPt (d) PdRh.

Washed cell suspensions of *Geobacter sulfurreducens* were first exposed to single-metal PGM solutions to constrain and identify the formation of different bio-PGM nanoparticles. When cells were challenged with Pd(ii) and supplied with acetate as electron donor, within 1 hour the colour of the solution changed from golden to grey/black (Fig. 1b), indicating the bioreduction of Pd(ii) to form Pd(0) nanoparticles.^8^ At 3 hours the cells had removed 80% Pd from solution, increasing to 89% removal at 24 hours (Fig. 1a). In the absence of cells, all Pd was removed from solution after 24 hours and a yellow precipitate had formed due to the hydrolysis of Pd(ii) at neutral pH, resulting in the precipitation of polynuclear Pd hydroxide complexes.^7,30^

The biorecovery of monometallic Pt and Rh by *G. sulfurreducens* was highly efficient, achieving more than 95% removal of each metal from solution within the first hour (Fig. 1a). In both samples, a rapid colour change from colourless to black was observed, indicating bioreduction of the PGMs to form metallic nanoparticles (Fig. 1b). In the absence of cells, no removal of aqueous Pt(iv) or Rh(iii) occurred, showing that only microbial processes were responsible for their removal from solution, and demonstrating the higher stability against hydrolysis in bicarbonate buffer at pH 7 of Pt(iv) and Rh(iii) compared to Pd(ii).

#### 3.1.2 Biorecovery from bimetallic PGM solutions

*G. sulfurreducens* cell suspensions, supplemented with acetate as an electron donor, were next challenged with bimetallic solutions containing 100 µM Pd(ii) and either Pt(iv) or Rh(iii). When cells were challenged with Pd(ii) and Pt(iv) together the yellow solution turned black within 1 hour, indicating bioreduction occurred. Cells removed Pt at a faster rate and to a greater extent than Pd; at 24 hours, cells had removed 95% Pt and 88% Pd from the bimetallic solution (Fig. 1c). The rate of Pt removal was significantly slower in the bimetallic solution compared to monometallic Pt; in the first hour, 50 µM of Pt (50%) was removed from the bimetallic solution, compared to 180 µM of Pt (98%) from the monometallic solution. The slower Pt removal by cells from the Pd–Pt bimetallic solution likely occurred because Pt(iv) and Pd(ii) compete for the same biosorption sites.^31^ In the Pd–Pt system without cells, metal removal matched the monometallic samples, with complete removal of Pd and no removal of Pt, indicating that Pd(ii) hydrolysis reactions did not impact Pt stability in solution.

In the bimetallic Pd–Rh system, rapid and near-complete removal of both metals from solution was observed, with 97% Rh and 85% Pd removed within 1 hour (Fig. 1d). Rh removal was comparable to monometallic Rh, but the rate and extent of Pd removal increased significantly compared to monometallic Pd and bimetallic Pd–Pt in the presence of Rh, achieving 95% removal by 24 hours (Fig. 1d). In the no cell control, the same gradual and complete removal of Pd occurred as in all samples, interestingly however, ∼20% Rh removal occurred in contrast to no removal in monometallic Rh, suggesting that Pd(ii) hydrolysis and precipitation led to partial Rh removal (Fig. 1d).

### 3.2 X-ray diffraction of biosynthesised PGM nanoparticles

**Fig. 2:**
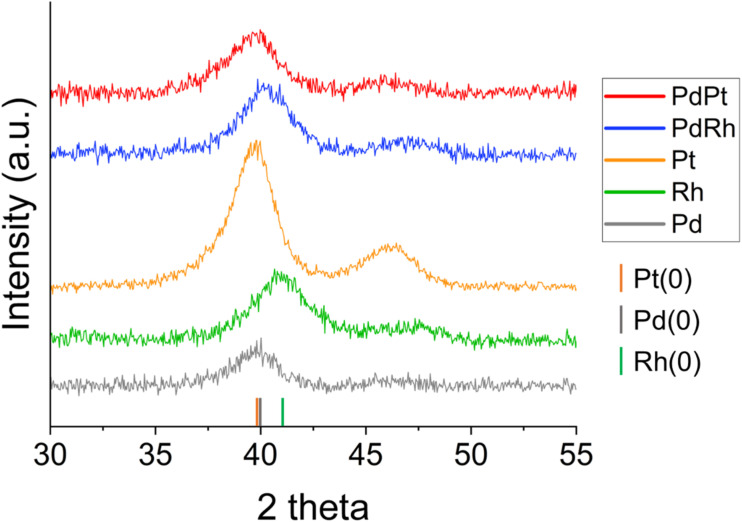
X-ray diffraction patterns for all PGM nanoparticles biosynthesised by *G. sulfurreducens*.

XRD was performed to understand the crystalline properties of the PGM nanoparticles biosynthesized by *G. sulfurreducens* after 24 hours. Analyses revealed that Pd(0), Pt(0) and Rh(0) were the only crystalline species present in monometallic bio-Pd, bio-Pt, and bio-Rh samples; in most cases, only cubic {111} planes gave a strong enough XRD peak for identification, due to the nanocrystalline nature of the samples (Fig. 2). Bragg equation indexing measured characteristic *d*-spacings of 2.251 Å for Pd(0), 2.200 Å for Rh(0), and 2.263 Å and 1.963 Å, indicative of the {111} and {200} planes, for Pt(0).^32^ These results confirm *G. sulfurreducens* bioreduced aqueous Pd(ii), Pt(iv) and Rh(iii) forming metallic nanoparticles, indicated by the colour change of the solutions to black. The Scherrer equation^33^ was used to calculate mean crystallite diameters, using the {111} peak for each metal, as 3.76 nm, 3.61 nm and 2.80 nm for Pd(0), Pt(0) and Rh(0), respectively (Table 2).

The {111} planes of bimetallic PdPt bionanoparticles possessed a *d*-spacing of 2.259 Å, which is comparable to a Pd–Pt metal alloy.^34,35^ Applying Vegard's Law^36^ to the {111} peak for bio-PdPt gave an alloy composition of Pd-40 Pt-60%. However, there is a large margin of error here in assigning an alloy from only one weak, broad nanocrystalline diffraction peak as the lattice parameters of Pd and Pt metals are very similar. The Scherrer equation mean crystallite diameter for bio-PdPt is 2.87 nm; smaller than that of bio-Pd or bio-Pt particles.

The measured *d*-spacing of the {111} planes of bimetallic PdRh bionanoparticles was 2.237 Å, which is in between measured monometallic Pd(0) and Rh(0) values and comparable to that of a Pd–Rh alloy.^37^ Applying Vegard's Law to the {111} peak for bio-PdRh gave an alloy composition of Pd-71 Rh-29%. The Scherrer equation using the {111} peak gave a mean crystallite diameter of 2.93 nm for bio-PdRh, smaller than that of bio-Pd and similar than the size of monometallic bio-Rh crystallites.

### 3.3 X-ray photoemission spectroscopy of biosynthesised PGM nanoparticles

**Fig. 3:**
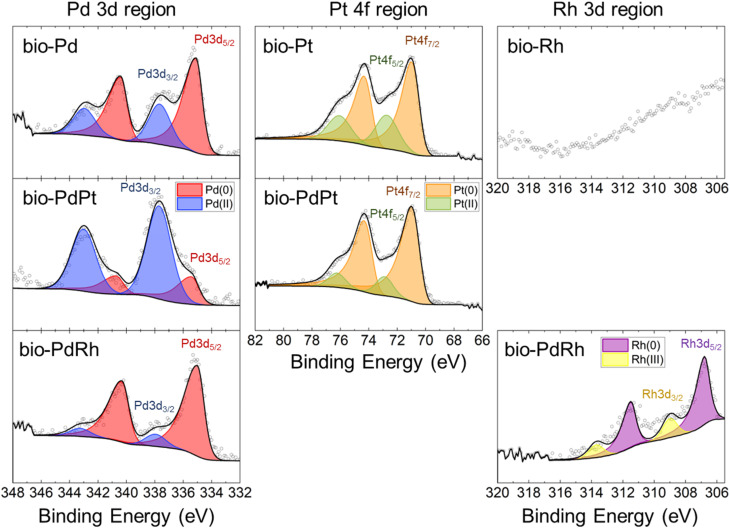
X-ray photoelectron spectroscopy results showing the fitting of high-resolution spectra for monometallic bio- Pd, Pt, and Rh, and for bimetallic bio- PdPt and PdRh nanoparticles. No XPS signal was detected for Rh in bio-Rh and fitting of the Rh 3d region was challenging due to high background from the nearby C 1s region peak.

XPS was used to identify the surface oxidation state of PGM bionanoparticles. Monometallic bio-Pd was comprised of Pd(0) and Pd(ii), with Pd(0) the dominant state (70%) (Fig. 3, and Table 1), in agreement with our previous work.^8^ In bio-Pt, Pt(0) was the dominant state (72%), but notably, no Pt(iv) was detected and Pt(ii) was the only oxidised surface state, indicating all Pt(iv) was reduced by the bacterium. In comparison, in bimetallic bio-PdPt the amount of Pt(0) increased to 86%, and Pd(0) decreased significantly to 23%, compared to monometallic bio-particles (Fig. 3, and Table 1). These results show that Pt(iv) is more favourably bioreduced by cells compared to Pd(ii) within a bimetallic Pd–Pt solution, which agrees with the higher percentage of Pt biorecovery observed. Previous work reporting XPS analysis of bimetallic bio-PdPt nanoparticles has found that particles comprised entirely of Pt(0) and mostly Pd(0) with only low levels of Pd(ii).^24,38^ However, these studies utilized 50 mM formate as electron donor, which is a potent abiotic reducing agent of Pt(iv) and Pd(ii).^24,38^

**Table 1:** Summary of XPS quantification of bio-PGM monometallic and bimetallic nanoparticles from high-resolution spectra of the Pd 3d, Pt 4f, and Rh 3d regions, detailing the binding energy (B.E) and relative atomic concentration of each species

Sample	Pd(0)		Pd(ii)		Pt(0)/Rh(0)		Pt(ii)/Rh(iii)	
	B.E (eV)	%Pd	B.E (eV)	%Pd	B.E (eV)	%Pt/Rh	B.E (eV)	%Pt/Rh
Pd	335.1	70	337.7	30	—	—	—	—
Pt	—	—	—	—	70.6	72	72.7	28
Rh	—	—	—	—	—	—	—	—
PdPt	335.4	23	337.7	77	70.6	86	72.8	14
PdRh	335.0	89	337.9	11	306.8	81	309.0	19

XPS of bimetallic bio-PdRh nanoparticles showed the surface to be highly concentrated in both Rh(0) and Pd(0), with minor amounts of Pd(ii) and Rh(iii) detected (Fig. 3, and Table 1). The Pd 3d signal from bimetallic bio-PdRh showed a relatively higher concentration of Pd(0) compared to monometallic bio-Pd (Fig. 3, and Table 1). Unfortunately, no discernible signal was generated from monometallic bio-Rh to compare to, likely due to high background from the nearby C 1s peaks (284.4 eV) and the low dispersion of bio-Rh on the cell surface, in contrast to the high dispersion of bio-PdRh nanoparticles.

### 3.4 Transmission electron microscopy of biorecovered PGM nanoparticles

#### 3.4.1 TEM of monometallic PGM nanoparticles

**Fig. 4:**
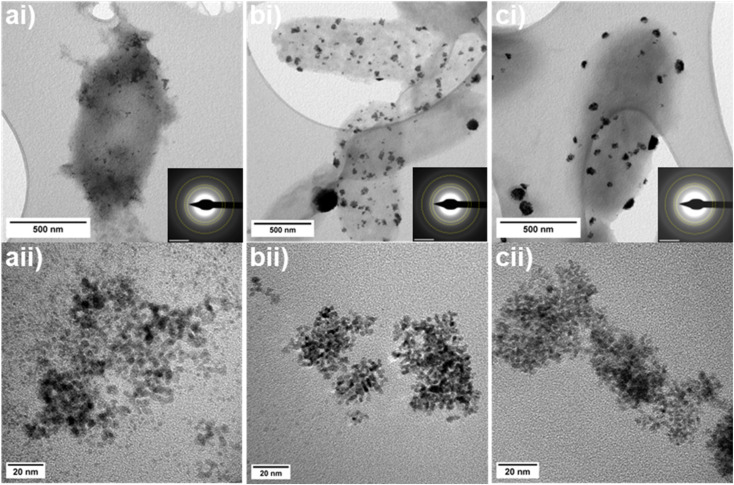
TEM images of biosynthesised PGM nanoparticles by *G. sulfurreducens* at (i) low and (ii) high magnification with SAED patterns inset (a) bio-Pd (b) bio-Pt (c) bio-Rh. Inset SAED scale bar is 5 1 nm^−1^, yellow dashed rings indicate the {111}, {200}, and {220} planes.

The morphology, distribution and particle size of the biosynthesised PGM nanoparticles formed was analysed using TEM. Selected area electron diffraction (SAED) patterns for bio-Pd, bio-Pt, and bio-Rh nanoparticles were indexed to the {111}, {200}, and {220} planes of metallic Pd(0), Pt(0), and Rh(0), respectively, in agreement with XRD data (Fig. 4a(i)–c(i)). The mean particle sizes of monometallic PGM nanoparticles were measured from high magnification TEM images as 3.76 ± 0.49 nm for bio-Pd, 3.28 ± 0.47 nm for bio-Pt, and 2.73 ± 0.45 nm for bio-Rh, which are broadly in agreement with XRD Scherrer equation results for crystallite size (Table 2). The distribution of the bionanoparticles varied between samples, with bio-Pd particles generally finely dispersed across the cell surface, but occasionally appeared as concentrated clusters of particles (Fig. 4a(ii)). In contrast, bio-Pt and bio-Rh nanoparticles only appeared as large, concentrated agglomerates, tens of nanometers in size, on the surface of *G. sulfurreducens* (Fig. 4b(ii) and 4c(ii)).

**Table 2:** Summary of particle size and crystallographic data from XRD and TEM analysis for PGM bionanoparticles

Sample	XRD mean crystallite size (nm)	TEM mean size (nm)	XRD {111} *d*-spacing	HRTEM {111} *d*-spacing	Reference {111} *d*-spacing	
Bio-Pd	3.76	3.76 ± 0.49	2.251	—	2.246	32
Bio-Pt	3.61	3.28 ± 0.47	2.263	—	2.265	32
Bio-Rh	2.80	2.73 ± 0.45	2.200	—	2.196	32
Bio-PdPt	2.87	2.61 ± 0.73	2.259	2.264	2.253	34
Bio-PdRh	2.93	2.39 ± 0.85	2.237	2.229	2.234	37

#### 3.4.2 TEM of bimetallic PGM nanoparticles

**Fig. 5:**
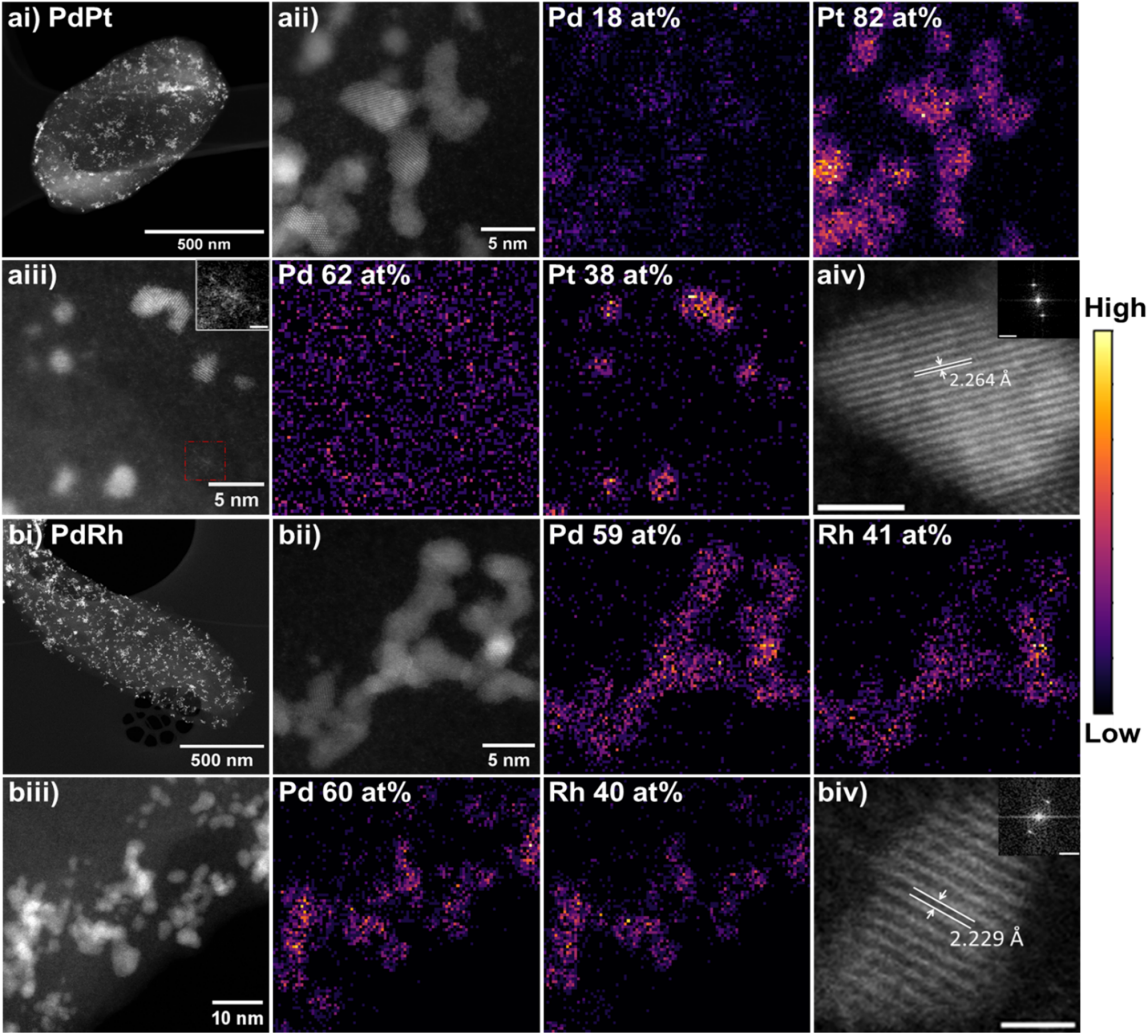
Bimetallic PGM nanoparticles biosynthesised by *G. sulfurreducens* (a) bio-PdPt (b) bio-PdRh. (i) Low magnification HAADF-STEM image of cell supporting bionanoparticles. (ii andiii) HAADF STEM images and EDS elemental mapping of the same area; at% quantifications given inset are an average over the whole spectrum image field of view. Inset of a(iii) highlighting presence of sub-nm clusters, scale bar 1 nm. (iv) High resolution TEM images of bimetallic nanoparticles showing atomic planes and their measured *d*-spacing; scalebar is 1 nm and inset FFT scalebar is 5 1 nm^−1^.

The size, morphology and elemental distribution of bimetallic PGM bionanoparticles were analysed with HAADF-STEM and EDS mapping. The mean particle sizes of bimetallic bionanoparticles, 2.61 ± 0.73 nm for bio-PdPt and 2.39 ± 0.85 nm for PdRh, were smaller than their monometallic counterparts. Both bimetallic bio-PdPt (Fig. 5a) and bio-PdRh (Fig. 5b) nanoparticles were finely dispersed across the surface of *G. sulfurreducens*, and were agglomerated in chains or clusters of particles (Table 2). Therefore these bimetallic particles more closely resembled the morphology of bio-Pd (Fig. 4a), as monometallic bio-Pt or bio-Rh (Fig. 4b and c) were deposited as large agglomerates of small nanoparticles. EDS mapping shows the extent of colocalization of the metals within the bimetallic nanoparticles. Quantification of the characteristic X-ray signals of Pd and Pt revealed that although the two metals often colocalized, bio-PdPt nanoparticles were far more highly concentrated in Pt (82 at%) compared to Pd (18 at%) (Fig. 5a(ii)). Additionally, analysis of Fast Fourier transforms (FFTs) of atomic resolution HAADF-STEM images of bio-PdPt nanoparticles revealed the *d*-spacing of the {111} atomic planes to be 2.264 Å, closely matching Pt(0) (Fig. 5a(iv)).^32^ EDS quantification of highly disperse bio-PdPt nanoparticles showed that these single nanoparticles were highly concentrated in Pt, whereas the cell surface (background) was highly concentrated in Pd (Fig. 5a(iii)). High resolution STEM imaging revealed the presence of sub-nanometre clusters supported on the cell surface, which are likely to be clusters of unreduced Pd(ii) (Fig. 5a(iii)), in agreement with our XPS results.

In contrast, EDS elemental mapping of bimetallic bio-PdRh nanoparticles shows co-localisation of the two metals, with quantification revealing concentrations of 60 at% Pd and 40 at% Rh in the nanoparticles (Fig. 5b(ii) and (iii)). In addition, FFT analysis revealed a *d*-spacing of 2.229 Å for bio-PdRh (Fig. 5b(iv)), which corresponds to the expected spacing for the {111} planes of a PdRh alloy.^37^

### 3.5 Bimetallic bionanoparticle synthesis mechanisms

#### 3.5.1 Bio-PdPt

Our results suggest that co-bioreduction of Pd(ii) and Pt(iv) led to a mostly heterogeneous bimetallic nanostructure, due to the preferential bioreduction of Pt(iv) over Pd(ii). Pt was more extensively removed from solution by cells and at a faster rate than Pd, the bio-PdPt surface was mostly comprised of Pt(0) and Pd(ii) according to XPS, and HAADF-STEM and EDS showed nanoparticles were mostly comprised of Pt(0) whereas sub-nm Pd clusters were identified at the cell surface, likely biosorbed Pd(ii). Additionally, any Pd(0) that is bioreduced can be galvanically displaced by Pt(iv)/Pt(ii) to form Pt(0), likely contributing to the very high dispersity of Pd(ii) clusters in bio-PdPt.

These results could be unexpected as the starting Pt(ii) and Pt(iv) chloro-complexes used in this study possess similar reduction potentials of 0.68 V and 0.62 V, respectively, which in general can yield alloy structures when co-reduced. However, we suggest that changes in metal speciation caused by solution chemistry and microbe-metal interactions alter the reduction potential of the metals, leading to the heterogeneous bimetallic nanostructure observed. The initial biosorption of negatively charged Pt(iv) chloro-complexes on bacterial surfaces was shown to result in non-enzymatic bioreduction to Pt(ii) chloro-complexes, likely through coordination to protonated amine groups.^39^ Our XPS results agree with this finding as only Pt(ii), and no Pt(iv), was detected in both monometallic bio-Pt and bimetallic bio-PdPt. Therefore the biosorption of Pt(iv)Cl_6_^2−^ to form Pt(ii)Cl_4_^2−^ would increase the reduction potential of Pt from 0.68 to 0.75 V. Conversely, PdCl_4_^2−^ immediately undergoes hydration to PdCl_3_(H_2_O)^−^ followed by hydrolysis to hydroxo–chloro complexes (such as [PdCl_3_(OH)]^2^ and [PdCl_2_(OH)_2_]^2−^) in bicarbonate buffer at pH 7,^7^ which lowers the reduction potential below 0.62 V *e.g.* Pd(OH)_4_^2−^ has a reduction potential of 0.1 V.^40^ The preferential bioreduction of biosorbed Pt(ii)Cl_4_^2−^ over Pd(ii) hydroxo–chloro complexes may therefore be explained by a large shift in their reduction potentials.

#### 3.5.2 Bio-PdRh

In contrast, the bioreduction of Pd(ii) and Rh(iii) by *G. sulfurreducens* led to the formation of PdRh alloy nanoparticles, according to lattice parameter measurements from XRD and high resolution HAADF-STEM. Vegard's Law applied to the XRD data suggested an alloy mixture of Pd-71% Rh-29%, and EDS quantification suggested an alloy composition of Pd-60% Rh-40%. These results suggest that under our reaction conditions the Pd(ii) and Rh(iii) complexes interacting with *G. sulfurreducens* may have possessed similar reduction potentials, as bimetallic alloy nanoparticles tend to form when two metal ions with similar reduction potentials are co-reduced in solution.^12^ Similarly to Pd(ii), Rh(iii) chloride complexes readily undergo hydration and hydrolysis reactions to form chloro-aqua-hydroxo species, making their speciation and reduction potential complicated and beyond the scope of this study.

### 3.6 Catalytic performance of biosynthesised PGM nanoparticles

**Scheme 1:**
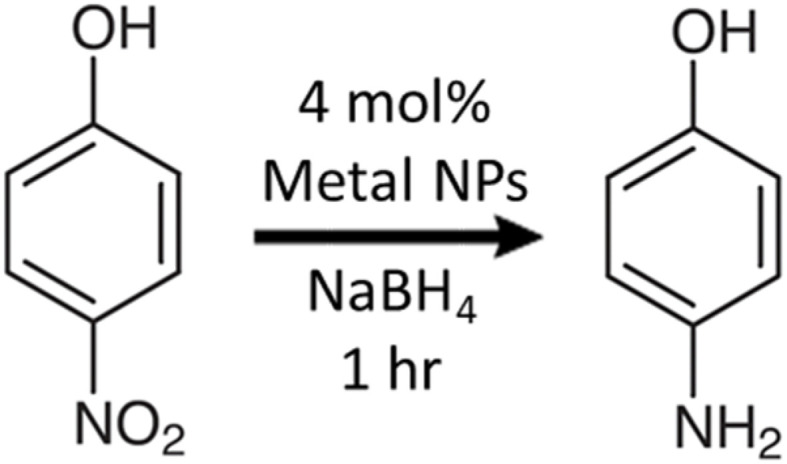
Reduction of 4-nitrophenol to 4-aminophenol.

The reduction of 4-nitrophenol to 4-aminophenol (Scheme 1) is a model hydrogenation reaction commonly used to benchmark the catalytic activity of metal nanocatalysts.^41^ The reaction occurs between the 4-nitrophenolate ion and H^+^ from NaBH_4_ when they are adsorbed onto the metal surface, following a heterogenous Langmuir–Hinshelwood mechanism.^42^

**Fig. 6:**
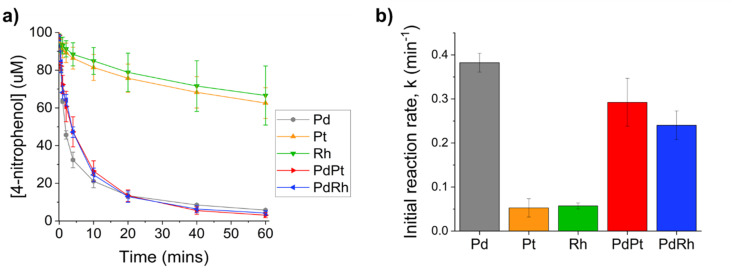
Reduction of 4-nitrophenol catalysed by biosynthesised monometallic and bimetallic PGM nanoparticles (a) decrease in 4-nitrophenol concentration over time (b) comparison of the initial reaction rate for each PGM catalyst. Catalyst loading for all reactions was normalised to 4 mol% total metal.

Monometallic PGM nanoparticles formed by *G. sulfurreducens* catalysed this reaction to different extents. Bio-Pd nanoparticles achieved 95% 4-nitrophenol reduction after 1 hour and gave the fastest initial rate of reaction (0.38 min^−1^) of all the biosynthesised nanoparticles (Fig. 6a and b). In contrast, bio-Pt and bio-Rh nanoparticles were significantly less active, only achieving 36% and 32% 4-nitrophenol reduction, respectively, after 1 hour (Fig. 6a). The superior catalytic performance of bio-Pd is not surprising as Pd is generally more effective in hydrogenation reactions compared to the other PGMs due to a lower hydrogen bonding energy, resulting in the optimal hydrogen adsorption and higher activity at lower temperatures.^43^ Indeed, comparison of Pd and Pt nanoparticles supported on mesoporous silica demonstrated Pd was far more active than Pt for 4-nitrophenol reduction.^44^ In addition, bio-Pd nanoparticles were more highly dispersed across the cell surface compared to bio-Pt and bio-Rh, which performed poorly despite their smaller particle size, likely due to the dense agglomeration of bio-Pt and bio-Rh nanoparticles leading to lower available surface area for reactants. In comparison, highly dispersed Pt nanoparticles supported on spherical polyelectrolyte brushes, and Rh nanoparticles supported on fullerene, have been reported to catalyse this reaction, under similar conditions as this study, completely in 20 and 10 minutes, respectively.^42,45^

Microbially synthesised bimetallic bio-PdPt and bio-PdRh nanoparticles displayed comparable 4-nitrophenol activity to bio-Pd while using approximately half the amount of Pd, and significantly outperformed monometallic bio-Pt and bio-Rh. Microbial bio-PdPt nanoparticles achieved 97% 4-nitrophenol reduction after 1 hour with an initial reaction rate of 0.32 min^−1^ (Fig. 6). In comparison, previous bio-PdPt nanoparticles synthesised using *S. oneidensis* produced an initial reaction rate of 0.031 min^−1^ using a 7.5 µM metal loading.^24^ As there was little evidence of Pd–Pt alloying the enhanced catalytic performance of bio-PdPt compared to bio-Pt is likely due to the highly dispersed sub-nm Pd(ii) clusters acting as a pre-catalyst, generating highly active Pd(0) clusters *in situ* upon reaction with NaBH_4_.^8^

Bio-PdRh alloy nanoparticles, synthesized for the first time *via* microbial bioreduction, achieved 96% conversion after 1 hour with an initial reaction rate of 0.25 min^−1^ (Fig. 6). The enhanced catalytic performance of bio-PdRh can be attributed to the biosynthesis of PdRh alloy nanoparticles, as alloying of two metals can change the surface energy and electronic state of nanoparticles compared to the parent metals, resulting in, for example, modified H binding strength, which can improve catalytic performance.^46^ PdRh alloys have shown promising catalytic performance for several important types of reactions, for example, reducing catalyst poisoning in the selective hydrogenation of quinoline,^20^ and demonstrating excellent conversion, selectivity and stability for the selective hydrogenation of 3-nitrophenylacetylene.^47^ Taken together, Table S1 shows that our biogenic catalysts are competitive with other bio and chemically-synthesised PGM benchmarks. The Pd-lean bimetallics retain high *k* relative to bio-Pd under surfactant-free conditions. Table S1 specifies dosing definitions and additives for each literature entry, with the higher *k* reports from the non-bio Pd particles relying on polymer or ligand stabilisers, oxide supports, or different normalisation bases. Future work should investigate the selectivity and poisoning resistance of biosynthesised PdRh alloy nanoparticles for a wider range of synthesis reactions.

## 4. Conclusion

This study presents new insights into the biosynthesis of bimetallic platinum group metal (PGM) nanoparticle catalysts by metal-reducing bacteria. We report the novel microbial biosynthesis using *G. sulfurreducens* of PdRh alloy nanoparticles through the co-bioreduction of Pd(ii) and Rh(iii). Under the same conditions using Pd(ii) and Pt(iv), bimetallic bio-PdPt particles were biosynthesised comprised of Pt-rich nanoparticles surrounded by biosorbed Pd(ii) clusters. These findings suggest that the reduction potential of metal complexes is critical in determining the nanostructure of mixed metal nanoparticles synthesised *via* outer membrane cytochrome-mediated bioreduction. The bimetallic bio-nanoparticles were found to possess far superior catalytic performance in a common hydrogenation reaction compared to their monometallic counterparts, apart from bio-Pd, which had the fastest initial reaction rate. Overall, this work contributes to our understanding of microbial metal bioreduction and nanoparticle formation from mixed metal solutions. The improved catalytic performance of mixed metal nanoparticles supports the development of microbial metal biorecovery and recycling of PGMs from mixed metal waste streams.

## Conflicts of interest

The authors declare no conflicts of interest.

## Supplementary Material

NA-008-D5NA00861A-s001

## Data Availability

The data that support the findings of this study are available from the corresponding authors: Prof. Jonathan R. Lloyd, Dr Jinxin Xie and Dr Christopher Egan Morriss. Supplementary information (SI): (Table S1) provides a literature comparison of apparent rate constants (*k*) for NaBH_4_-driven 4-nitrophenol reduction using PGM catalysts, including reaction conditions and references. See DOI: https://doi.org/10.1039/d5na00861a.
