# Supplementary material for: Microbial synthesis of bimetallic Pd–Rh and Pd–Pt nanoparticle catalysts
Source: Nanoscale Adv. 2025 Dec 16;8(3):961–72. doi: 10.1039/d5na00861a (PMC12713794; doi:10.1039/d5na00861a)
Supplement: NA-008-D5NA00861A-s001 [file NA-008-D5NA00861A-s001.pdf]

Supplementary Information (SI) for Nanoscale Advances.
This journal is © The Royal Society of Chemistry 2025

Table S1 Apparent rate constants for 4-nitrophenol reduction with $NaBH_4$ (PGM catalysts only)；RT: Room temperature

**Catalyst / system**	**k(min^{-1})**	**Dose basis**	**Medium and additives**	**Temp**	**Source**
This work - bio-Pd	0.38	4 mol% total PGM vs 4-NP	Water, surfactant-free	RT	This work
This work - bio-PdPt (Pt-enriched)	0.32	4 mol% total PGM vs 4-NP	Water, surfactant-free	RT	This work
This work - bio-PdRh (alloyed)	0.25	4 mol% total PGM vs 4-NP	Water, surfactant-free	RT	This work
Bio-Pd, *Shewanella oneidensis*	0.0142	7.5 μM Pd in reactor	Water	RT	(Tuo et al 2017)
Bio-Pt, *Shewanella oneidensis*	0.0056	7.5 μM Pt in reactor	Water	RT	(Tuo et al 2017)
Bio-PdPt, *Shewanella oneidensis*	0.0292	7.5 μM Pd+Pt in reactor	Water	RT	(Tuo et al 2017)
Bio-Pd, *Shewanella oneidensis* (AQDS)	0.0256	7.5 μM Pd in reactor	Water	RT	(Tuo et al 2017)
Bio-Pt, *Shewanella oneidensis* (AQDS)	0.0065	7.5 μM Pt in reactor	Water	RT	(Tuo et al 2017)
Bio-PdPt, *Shewanella oneidensis* (AQDS)	0.0316	7.5 μM Pd+Pt in reactor	Water	RT	(Tuo et al 2017)

Pd NPs, PVA-stabilised	0.514	2.91 mol% Pd vs 4-NP	Water, PVA stabiliser	298 K	(Chatterjee and Bhattacharya, 2021)
Pt NPs, rhodizonate-stabilised	0.1088	-	Water, sodium rhodizonate	RT	(Islam et al., 2018)
Pd NPs, rhodizonate-stabilised	0.0165	-	Water, sodium rhodizonate	RT	(Islam et al., 2019)

Reference:

Tuo, Y., Liu, G., Dong, B., Yu, H., Zhou, J., Wang, J., & Jin, R. (2017). Microbial synthesis of bimetallic PdPt nanoparticles for catalytic reduction of 4-nitrophenol. E*nvironmental Science and Pollution Research*, 24(6), 5249-5258.

Chatterjee, S., & Bhattacharya, S. K. (2021). Size-dependent catalytic activity of PVA-stabilized palladium nanoparticles in p-nitrophenol reduction: using a thermoresponsive nanoreactor. *ACS omega*, *6*(32), 20746-20757.

Islam, M. T., Saenz-Arana, R., Wang, H., Bernal, R., & Noveron, J. C. (2018). Green synthesis of gold, silver, platinum, and palladium nanoparticles reduced and stabilized by sodium rhodizonate and their catalytic reduction of 4-nitrophenol and methyl orange. *New Journal of Chemistry*, *42*(8), 6472-6478.
